# Assessing Day-to-Day Emotion Dynamics Within the Whole Family: Protocol for a Family-Wide Ecological Momentary Assessment Study (The Family and Child Emotion Study)

**DOI:** 10.2196/77364

**Published:** 2025-10-06

**Authors:** Natasha Vogel, Linda Sosa-Hernandez, Charlotte Funston, Evelyn Balfour, Kristel Thomassin

**Affiliations:** 1 Department of Psychology University of Guelph Guelph, ON Canada

**Keywords:** ecological momentary assessment, family, child, emotion, physiology, regulation

## Abstract

**Background:**

Families play a pivotal role in shaping children’s emotional development through emotion socialization. However, most research has focused on individual or dyadic relationships, such as those between parents and children, overlooking the more complex dynamics that emerge when multiple family members interact simultaneously. This limited perspective fails to capture the full scope of the interconnected emotional processes within family units. A contributing factor to this gap is the limited availability of models suited for capturing and analyzing complex, family-level data.

**Objective:**

The Family and Child Emotion Study aims to address this gap by examining family-wide emotion dynamics across all family members—including parents and children—as they naturally unfold in daily life.

**Methods:**

This protocol uses a pre-post design and a 7-day ecological momentary assessment period combined with ambulatory monitoring of heart rate and electrodermal activity within whole family units to examine interactions among mothers, fathers, and siblings, providing a comprehensive understanding of family-wide emotion processes. Data will be analyzed using a network analytical approach, specifically multilevel vector autoregressive modeling, to investigate dynamic emotional processes within and between family members.

**Results:**

Funding was received in April 2020. Data collection began in September 2022 and will continue until March 2026. As of May 2025, the Family and Child Emotion Study has collected data from 48 eligible families. Data analyses will begin after March 2026, with results expected to be published in Fall 2027.

**Conclusions:**

This study introduces an innovative approach for examining emotion dynamics within whole-family systems in naturalistic settings, offering practical guidance for collecting and analyzing complex, multilevel, and nested data. The primary aim is to investigate how family emotion networks contribute to children’s emotional functioning and development. A secondary aim is to explore key factors, such as parental psychological functioning and child emotion regulation abilities, that may shape these networks. This protocol serves as a valuable framework for future researchers exploring family-wide emotion dynamics.

**International Registered Report Identifier (IRRID):**

DERR1-10.2196/77364

## Introduction

### Background

Emotions are central to our behaviors, social interactions, and daily functioning. Key emotional skills, such as recognizing, labeling, and regulating emotions, are crucial for optimal functioning within society. Children who struggle with emotion regulation are at a higher risk for a range of negative outcomes, including academic and social difficulties, impaired executive functioning, and psychopathology [1-5]. Therefore, understanding the processes that foster healthy emotion development in children is essential for promoting positive health and well-being. A robust body of literature identifies the family, and particularly parents and caregivers (hereafter “parents”), as a primary influence in shaping children’s emotional development through emotion socialization [6-8]. Emotion socialization is the process through which children learn how to understand, express, and regulate emotions, primarily through interactions with caregivers, family members, and broader social contexts [6]. Most research on emotion socialization to date has focused on mother-child interactions. In recent years, research on emotion socialization has expanded to include fathers and siblings, yet still to a much lesser degree than research with mothers. Scarce research has captured more complex dynamics such as triadic family interactions involving multiple family members simultaneously [9-12]. As emotion socialization frequently occurs through subtle and indirect processes within the whole family (eg, via modeling and indirect teaching), research designs must account for these nuanced processes and consider how multiple family members influence one another in dynamic and interconnected ways [6,13].

Family Systems Theory revolutionized our understanding of families, moving beyond the individual and mother-child dyads that dominated the focus of research at the time [14,15]. Family Systems Theory posits that the family unit is more than a sum of its parts, but rather a complex system comprised of multiple subsystems—ranging from individuals to dyads and triads—that are interdependent and dynamically interact [14,15]. These subsystems engage in ongoing transactional and bidirectional exchanges, suggesting that isolating any single dyadic relationship provides an incomplete view of the family unit’s functioning. The emphasis on individual or dyadic relationships in earlier emotion socialization research likely arose, in part, from methodological and statistical constraints, including challenges associated with collecting, managing, and analyzing complex family-level data. Technological advancements in data collection methods, such as physiological monitoring, ecological momentary assessment (EMA), and advanced statistical modeling techniques, including multilevel modeling and network analyses, now enable more sophisticated approaches to capturing the interconnected nature of family relationships in everyday life, providing greater ecological validity and adding nuance to family-based research. Despite these developments, no existing studies have examined emotion socialization at the whole-family level, limiting the dissemination of research protocols that can be used widely. In this paper, we present a protocol for the Family and Child Emotion Study (FACES), which examines real-world emotion dynamics within entire family units, including interactions among mothers, fathers, children, and their siblings. The study uses a pre-post design and a 7-day EMA period paired with ambulatory monitoring of heart rate (HR) and electrodermal activity (EDA) across all family members within a household. This research aims to deepen our understanding of how emotions are experienced, expressed, and regulated within family systems in daily life. Furthermore, this protocol provides a prototype for how such research can be designed.

### Dynamic Emotion Exchanges Within the Family System

Family emotion dynamics encompass the patterns and trajectories through which aspects of emotional experiences—such as experiential, physiological, and behavioral components—fluctuate and interact among family members over time [16,17]. From a Family Systems perspective, these dynamics function primarily to maintain or restore homeostasis within the family unit [14,15]. As such, emotion exchanges are a key mechanism for emotion socialization within the family. Emotion socialization can occur in direct ways, such as when parents teach children about emotions and emotion expression, as well as indirect ways, such as when parents model emotion-related behaviors and react to children’s expression of emotion [6]. Indirect emotion socialization may also occur through spillover effects from one dyad to another (ie, Spillover Hypothesis), where the emotional interactions between one dyad influence another [18]. For instance, emotions displayed during coparent interactions can affect their child. Similarly, emotional contagion occurs when emotions are indirectly transferred from one individual to another or to the entire group [19,20]. Taken together, family-emotion dynamics are complex and encapsulate many indirect modes of socialization that are thought to contribute to children’s emotional development. However, studying these family-wide emotion dynamics requires accounting for several methodological and analytical factors during study design, implementation, and analysis.

### Research Design and Methodological Considerations in Studying the Whole-Family Unit

#### Overview

Studying family-emotion dynamics requires a research design that balances ecological validity, multilevel emotion measurement, and advanced analytical methods. Each of these components is essential for capturing the complex and interconnected emotional interactions that occur within whole-family systems.

#### Ecological Validity

Ecological validity refers to the extent to which research findings can be generalized to real-world settings [21]. Ecological validity is essential for understanding family-emotion dynamics because emotional interactions within families are influenced by complex and context-specific factors that may not manifest in artificial or controlled environments such as a research laboratory. Knowing that emotional processes are shaped not only by the members themselves but also by the surrounding environmental context, studying families in their real everyday environments yields meaningful information. EMA strengthens ecological validity by facilitating real-time data collection of participants’ experiences in their natural environments, such as at home or during daily routines [21,22]. The real-time assessment of emotions reduces retrospective bias and enhances the accuracy of reported emotions [23,24]. Furthermore, EMA can offer an additional layer of context by incorporating questions about participants’ current activities, social interactions, and environmental factors, offering deeper insight into emotional experiences.

#### Emotion Measurement

Emotions are complex responses to specific events or stimuli, encompassing subjective experiences, physiological reactions, and behavioral responses [25]. Thus, to fully understand family-wide emotion dynamics, it is essential to examine emotional experiences across multiple levels. Many studies focus on the experiential aspects of emotion through self-report measures, which provide valuable insights into individuals’ subjective emotional states. However, self-reports alone cannot capture other critical components of the emotional experience, such as physiological responses. Physiological aspects of emotion reflect internal processes regulated by the autonomic nervous system, including changes in HR and EDA (ie, bodily perspiration), both of which are strongly linked to emotional arousal, making them essential metrics for assessing emotional responses [26-29]. Measuring physiology in response to emotional experience necessitates the use of equipment capable of capturing the subtle, dynamic changes regulated by the autonomic nervous system. In-laboratory studies permit a wide range of tools used to collect physiological signals through sensors or electrodes placed on the body. However, capturing physiological responses in everyday life requires wearable ambulatory equipment that is both portable and nonintrusive. These devices must allow participants to carry out their daily activities while continuously recording physiological data. Examples of these types of technologies include wearable wristbands that either measure HR and EDA or just HR, as well as chest straps that measure HR. When studying emotion across families, it is crucial that assessment tools and equipment are suitable for individuals at different developmental stages, with equipment needing to be validated for use across age groups and appropriately sized for children [30]. A key advantage of ambulatory physiological monitoring is that it can be combined with EMA surveys, enabling the concurrent measurement of subjective emotional experiences and physiological arousal. In a family-wide context, fully understanding emotional processes requires the simultaneous collection of data from all family members to capture dynamic, real-time interactions. This approach allows researchers to investigate how emotions influence or align across family members. To achieve this, active participation from each family member is crucial, including completing emotional self-reports and having their physiological responses monitored.

#### Analytical Approach

The approaches described above yield numerous data points (eg, repeated surveys each day for several days), at multiple levels of measurement (eg, emotion self-report and physiology), for several family members, resulting in multiple levels of nested data captured repeatedly over an extended time frame. Thus, the analytic approach used for this type of data must account for the nested and interdependent nature of the data, as well as the longitudinal, repeated measurement design. Further, as with any intensive data collection design, approaches to missing data should be considered carefully. Other factors to consider include the synchrony between different family members’ reports, synchrony in emotion reports (self-reported) and physiology obtained passively through a wearable device, and the directionality of effects. Finally, a whole-family analysis needs an approach that can generate family-level and person-specific values to allow for further testing of individual differences in families (eg, how emotionally cohesive a family is) and family members (eg, the extent to which fathers influence the family unit). Taking all of these factors into account, we recommend a network analytic approach, specifically multilevel vector autoregressive modeling [31-33]. As will be described further in the analytic section, resulting family-emotion networks will provide a measure of density, or how connected the family emotion network is, and a measure of centrality for each family member, measuring how influential the member is within the whole family unit. By capturing the dynamic interplay of emotional and physiological processes within family systems, these networks provide a deeper understanding of how emotions are expressed and regulated during everyday family interactions. This approach offers unique insight into the emotional climate of the family and the roles individual family members play in shaping shared emotional dynamics. Given that emotion regulation is a key predictor of mental health and well-being [4], understanding how families influence each other’s regulation and emotion expression may offer insights into how family dynamics relate to psychological health and well-being.

### FACES

The FACES project offers a novel approach to studying real-world emotion dynamics within whole-family units by combining an EMA research design with ambulatory physiological monitoring. Unlike the majority of existing research focusing on specific dyadic relationships, FACES tracks experiences of emotion within whole-family units, with the capacity to assess multiple dyadic, triadic, and whole-family interactions in families’ daily interactions. The combination of EMA paired with ambulatory physiological monitoring is innovative and addresses calls for emotion measurement at multiple levels [17]. The primary objective of this protocol is to understand how family-emotion networks contribute to children’s functioning and psychological health. Moreover, the networks will disentangle the contributions of specific family members to child outcomes. A secondary objective of the research is to understand which family, parent, and child factors may influence family-emotion networks (eg, parental psychology and child emotion regulation abilities). Finally, the project focuses on children in middle childhood and early adolescence, as these are important periods of meaningful emotional development [34]. During this period, children experience substantial advancements in their ability to identify, express, and regulate emotions, as well as in their understanding of others’ emotions [34]. These skills form the foundation for effective social interactions and long-term emotional well-being. Research has shown that children as young as 7 years old can also participate independently in EMA research, providing reliable and valuable insights into their subjective emotional experiences [35]. By leveraging these methodological and analytic advances, FACES provides a comprehensive framework for capturing how family emotional dynamics unfold in daily life and how they shape children’s emotional development in meaningful and lasting ways. Thus, the aim of this paper is to describe the FACES study protocol, including its design, recruitment procedures, measures, and planned analyses to support implementation, enhance reproducibility, and serve as a guide for researchers examining emotion dynamics within family systems. This protocol was reported in accordance with the SPIRIT (Standard Protocol Items: Recommendations for Interventional Trials) guidelines. The completed SPIRIT checklist is provided in Multimedia Appendix 1.

## Methods

### Study Design and Setting

The FACES protocol uses a repeated-measures design consisting of a 7-day EMA period, combined with a pre- and poststudy framework. Conducted entirely remotely from families’ homes, this study integrates web-based surveys, daily self-reports of emotional experiences, and ambulatory monitoring of HR and EDA. Families are recruited from the local community and surrounding areas.

### Recruitment

Families who express an interest in participating are invited to complete a screening phone call to determine their eligibility. Recruitment relies on community self-referral through advertisements via social media and flyers around the community, as well as through in-person outreach (eg, booths at community centers and local family-focused community events).

### Eligibility Criteria

Families are considered eligible to participate if they meet all inclusion criteria and none of the exclusion criteria. To align with the study’s focus on whole-family emotion dynamics, a minimum of two parents and two children is required for participation. A parent is defined as an adult who has cohabited with their children for at least 2 years and serves in a caregiving role. This inclusion criterion allows for the examination of emotion processes not only at the individual level but also within dyadic (eg, parent-child and siblings), triadic, and full-family interactions. Including multiple family members is essential to capture the complex, interconnected nature of emotional experiences within families. Participating children should have no more than a 4-year age gap, and both children must be between the ages of 7 and 14 years. All family members must be proficient in reading, speaking, and comprehending the English language. Families are ineligible if the participating children have any developmental delays, such as delays in speech, hearing, or sensory integration. Based on well-known influences on vagal activities, any history of cardiovascular health problems and any medications that any participating family member uses other than over-the-counter analgesics or oral contraceptives are taken note of [36].

### Data Collection Procedures and Methods

#### Overview

**Figure 1:**
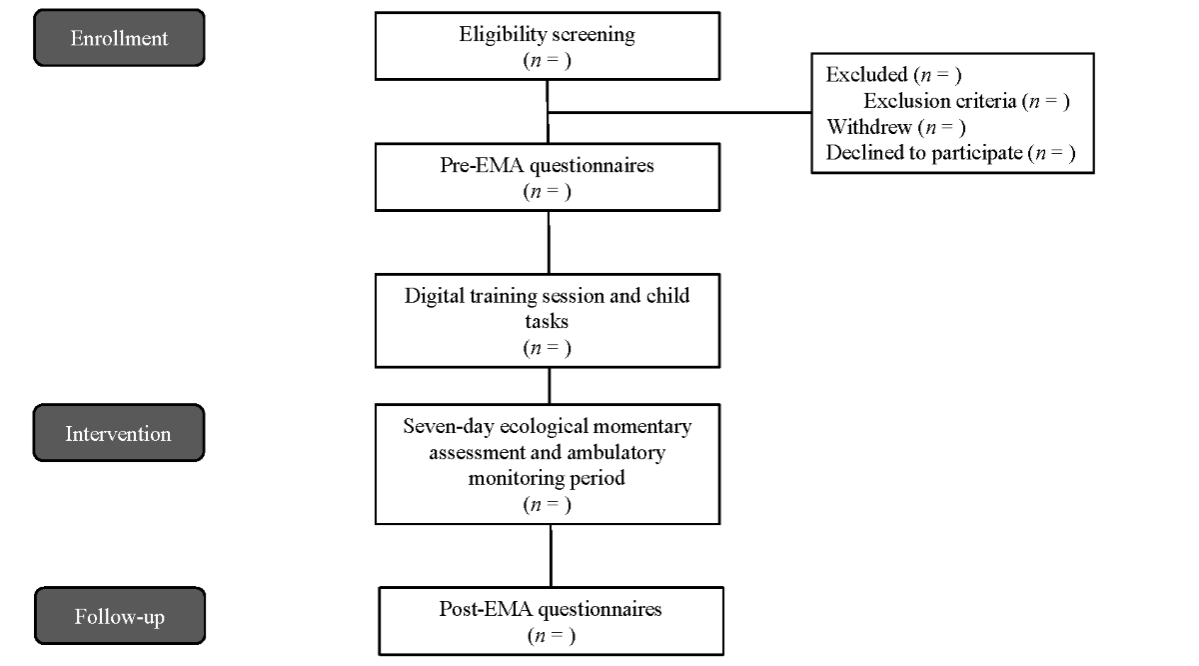
Consort diagram of participant flow. EMA: ecological momentary assessment.

Families begin by completing web-based questionnaires on Qualtrics (Qualtrics International Inc) sent via email, with a separate survey link provided for each family member. Once all participating family members have submitted the initial questionnaires, a research assistant drops off the study equipment (ie, phones, wristbands, chargers, and a laptop if needed) at the families’ home. To ensure proper use and care of the study equipment, families attend a training session with a research assistant via Teams (Microsoft Corp). During this session, children also complete additional tasks on the Teams call. The day following the training session, families begin the 7-day EMA and ambulatory monitoring period using the Time2Feel app (Troon Technologies) and Empatica E4 wristbands [37]. At the end of the study period, a research assistant picks up the equipment from the families’ homes, and family members complete final web-based questionnaires. In each family, one child aged 8 to 12 years is randomly selected to be the “focus child.” Figure 1 gives an overview of the participant timeline.

#### Pre-EMA Questionnaires

Each family member completes their own survey based on their role in the family (eg, one survey for each parent, one for the focus child, and one for the sibling). Separate links to these surveys are sent to each family member by email so they can complete their surveys independently. One of the parents provides demographic information about themselves, the other parent, and their children, including details such as age, gender, ethnicity, education level, occupation, medical diagnoses (physical and psychological), and household income. Both parents complete questionnaires that evaluate parenting practices, as well as their own and the focus child’s emotion regulation. Children complete questionnaires that assess emotional functioning, including emotion-regulation abilities. All survey items are presented in a fixed order, with no randomization or adaptive questioning. Page breaks are inserted after each measure, and the number of questions per page varies depending on the length of the measure. If a participant leaves a question blank, a prompt appears notifying them that some questions have not been answered, giving them the option to return and complete them or continue regardless. Once a participant advances to the next page, they cannot return to previous pages to change their responses. Completing parent questionnaires takes approximately 40 minutes, while the child questionnaires take about 20 minutes. A member of the research team reviews each survey for completeness before scheduling the training session. If entire scales are missing from a survey, the research team follows up by emailing the family to confirm whether the missing responses were intentional or due to an error. Duplicate survey entries are identified and manually reviewed using the participant ID. If a duplicate survey contains missing sections that are completed in another entry, the responses are combined. Otherwise, the first complete entry is used for analysis. Both completed and partially completed questionnaires are included in analyses, depending on the extent and pattern of missing data. If participants complete only part of the survey but provide responses to key measures relevant to specific research questions, their data are retained for those analyses. Completion time is monitored through Qualtrics, but no strict cut-off is used to exclude responses based on duration alone. Instead, potentially invalid submissions (eg, extremely short completion times coupled with missing data) are reviewed on a case-by-case basis to determine inclusion.

#### Training Session and Child Tasks

Parents and children participate in a training session via Teams, led by a graduate or senior undergraduate research assistant. During this session, the research assistant explains the EMA period, including how to use the Time2Feel phone app, and provides step-by-step instructions for setting up and pairing the Empatica E4 wristbands with the app. Participants also calibrate their wristbands with the app to establish baseline measurements for HR rate and EDA. The focus child then completes a computer-based cognitive reappraisal task (CRT) [38].

#### EMA Period

##### Overview

For 7 days, parents and children complete between 4 and 8 brief Time2Feel surveys and wear their assigned Empatica E4 wristband each day from 4 PM to 8 PM on weekdays and 10 AM to 6 PM on weekends. The duration of the EMA period was chosen to strike a balance between collecting a sufficient number of observations for network analyses while also minimizing participant burden, as some studies have found that compliance with EMA protocols tends to decline over the course of longer study periods [39]. Additionally, EMA prompts were scheduled to occur outside of school and typical work hours to better align with parents’ and children’s daily routines, as recommended by Sosa-Hernandez et al [30].

##### Time2Feel

Each survey contains four questions for children and five questions for parents. The first question asks, “How are you feeling right now?” where users are prompted to select their current emotional state from a list of 13 emotions: happy, nervous, sad, excited, relaxed, frustrated, scared, angry, surprised, proud, ashamed, guilty, and disgusted. The second question asks users to rate the intensity of their selected emotion on a slider from 0=very slightly or not at all to 100=extremely. The third question asks, “What are you doing right now?” with 12 activity options, including homework, cooking, chores, shopping, eating, playing games, work, exercising, watching television, relaxing, social media, and sports or outdoor activities. Users then indicate who they are with, selecting from 8 to 9 options (children: alone, mom, dad, brother, sister, whole family, friend, and group of friends; parents: alone, significant other, younger son, older son, younger daughter, older daughter, whole family, friend, and group of friends). Parents receive one additional question asking, “How stressed are you feeling right now about parenting?” where they rate their parenting stress on a sliding scale ranging from 0=very slightly or not at all to 100=extremely. Surveys are distributed four times daily using a stratified random-interval schedule, with one survey sent randomly in each of four time blocks. Participants may also receive up to four additional surveys triggered by a 15%-20% increase in baseline HR or EDA [40].

##### Empatica E4 Wristbands

The E4 wristbands are a certified medical wrist-worn device that collects real-time continuous physiological signals, including HR and EDA [41]. Each family member wears an E4 wristband for the allotted study hours of the 7-day EMA and ambulatory monitoring period. The Empatica E4 wristband is a validated tool for collecting HR and EDA data in both adult and youth samples [41]. Each wristband contains four sensors: photoplethysmography (PPG), EDA, infrared thermophile, and a 3-axis accelerometer [42]. HR is measured through the PPG sensor that detects changes in blood volume pulse, which is then translated into HR and interbeat intervals [42]. Although PPG is not considered the “gold standard” compared to electrocardiography (ECG), research indicates that HR recordings from the Empatica E4 are comparable to ECG measurements when movement is minimal [41].

EDA is measured using two electrodes that sit against the inside of the wrist that pass a current between the two electrodes to capture fluctuating changes in the electrical properties of the skin as measured in microsiemens (μS) at a sampling frequency of 4 Hz [42]. Though the wrist is not considered a “gold-standard” location to measure EDA, as there is a lower density of sweat glands compared to other sites (ie, palmar surface), it is much more practical and comfortable for the user and arguably provides an ideal signal-to-noise ratio when considering how often people use their hands in everyday activities [43]. The wristband is worn on the participants’ nondominant hands, snugly, to minimize motion artifacts. Each wristband was connected to the participants’ Time2Feel app via Bluetooth.

##### Post-EMA Questionnaires

Following the 7-day EMA and ambulatory monitoring period, parents and children complete a final web-based survey in the same format as the initial questionnaires. Post-EMA questionnaires ask about their experiences using the Time2Feel app and their own emotion-regulation practices. Additionally, parents are asked to report on the focus child’s emotion-regulation practices.

### Strategies to Improve Adherence and Retention

Based on recommendations from Sosa-Hernandez et al [30], a training session is conducted to promote adherence and compliance with the study protocol. The orientation provides information about study procedures, demonstrates how to use study equipment, and offers families an opportunity to ask questions. During the EMA period, a research assistant is available via email or phone to provide technical support and answer any additional questions as needed. Families receive a check-in message on the third day of their EMA period via email or text, providing an update on their progress toward the bonus and addressing any challenges that have been noted by the research team. As an additional retention strategy, birthday and thank you cards are mailed to families’ home addresses [44].

### Ethical Considerations

The FACES study was reviewed and approved by the University of Guelph’s Research Ethics Board (REB#19-07-018), in accordance with guidelines for research involving human participants. Informed consent is collected from each member of the family. Families complete a web-based consent form via Qualtrics prior to completing the initial questionnaires. The consent forms outline what each family member will take part in over the study period, the purpose of the study, the activities each family member will participate in, how long each activity will take, privacy and confidentiality, contact details for the primary investigator, and details about compensation. By consenting, family members agree to take part in study activities and data collection. However, consent can be withdrawn at any time or at any stage of the study without penalty, and any family member has the right to initiate withdrawal from the study. If any family member wishes to discontinue participation, the whole family is removed from the study. Participants are free to skip any questionnaire items they prefer not to answer and may opt out of specific study components that make them uncomfortable. For example, a child with sensory challenges may choose not to wear the Empatica E4 device for physiological data acquisition. In the demographic questionnaire, questions include nonresponse options (eg, “Prefer not to answer”), as well as an “Other” option with space to further specify. The initial web-based questionnaires use a closed survey format, meaning participants can only access the survey via a link sent by the research team. To begin the survey, participants are required to enter an ID provided by the researcher. Data collected through Qualtrics—including any personal information (eg, names and email addresses)—are securely downloaded and stored on a password-protected server within an encrypted network. Access to this data is restricted to authorized research team members, each using individual login credentials. Once downloaded, all data are deidentified, retaining only the family ID and participant role (eg, mother and father). Survey data and raw physiological data collected via the Time2Feel platform are stored on the protected server with family IDs and participant roles. Each family’s survey responses and Time2Feel reports are stored separately and are not accessible to other family members. Any video recordings from Teams are immediately transferred to a secure server, and all files are promptly deleted from Teams after transfer. The University’s secure server performs automatic, system-wide backups every night to ensure data integrity. Limits to confidentiality include potential disclosures involving potential child abuse or significant risks of harm to oneself or others. In such cases, the research team is legally required to take appropriate actions to ensure safety. This may involve reporting the information to the principal investigator, relevant authorities, or individuals who may be at risk. Families receive financial incentives after various stages of the study: the pre-EMA questionnaires, the EMA and ambulatory monitoring period, and the post-EMA questionnaires. Parents are compensated with CAD $20 (US $14.42) in gift cards for completing the pre-EMA questionnaires, CAD $50 (US $36.06) for participating in the EMA and ambulatory monitoring period, and CAD $5 (US $3.61) for completing the post-EMA questionnaires. Children receive CAD $5 (US $3.61) for completing the pre-EMA questionnaires, CAD $15 (US $10.81) for the EMA and ambulatory monitoring period, and CAD $5 (US $10.82) for the post-EMA questionnaires. Participants can also earn a CAD $5 (US $3.61) bonus for completing 80% or more of the EMA surveys.

### Outcomes

A comprehensive list of all measures and outcomes is presented in Table 1.

**Table 1:** List of study measures.

Outcome and measure				Type of report		Timepoints
**Family-wide outcomes**						
	User experience and emotion reports					
		Time2Feel emotion surveys	Self-report		EMA^a^	
		Empatica E4 wristband	Physiology		EMA	
		Usefulness, Satisfaction, and Ease-of-Use Questionnaire	Self-report		Post	
	Family functioning					
		Family Functioning Styles Scale	Parent-report		Pre	
**Parent outcomes**						
	Emotion socialization					
		Coping with Children’s Negative Emotions Scale	Self-report		Pre	
	Gendered emotion beliefs					
		Parents’ Gendered Emotion Beliefs Scale	Self-report		Pre	
	Emotion regulation					
		RESS^b^	Self-report		Pre	
	Negative emotionality					
		CADS^c^ (Adapted)	Self-report		Pre	
	Psychopathology					
		Kessler 10	Self-report		Pre	
	Perceived parental stress					
		Parental Stress Scale	Self-report		Pre	
**Child outcomes**						
	Emotion regulation					
		RESS	Parent and child report		Pre, post^d^	
		Emotion Regulation Checklist	Parent report		Pre, post	
		Cognitive reappraisal task	Child task		EMA	
	Negative emotionality					
		CADS	Parent and child report		Pre, post	
	Psychopathology					
		Brief Problems Monitor	Parent and child report		Pre, post	

^a^EMA: ecological momentary assessment.

^b^RESS: Regulation of Emotion Systems Survey.

^c^CADS: Child and Adolescent Disposition Scale.

^d^Parent-report on the RESS is completed only during the pre-EMA questionnaire, while the child report is completed at both pre- and post.

### Primary Outcomes: Engagement and Family Outcomes

#### Time2Feel Engagement and Usability

To assess the feasibility and usability of the EMA protocol, engagement and compliance with Time2Feel for each family member is calculated as the percentage of completed surveys out of the total survey prompts received during the 7-day EMA and ambulatory monitoring period. A survey is considered complete if all questions are answered, while incomplete responses have at least one missing answer. Separate response rates are calculated for both random and deviation surveys.

After the 7-day EMA and ambulatory monitoring period, each family member completes the satisfaction and ease-of-use and subscales from the Usefulness, Satisfaction, and Ease of Use questionnaire to assess their experiences using the Time2Feel app [45]. The satisfaction subscale includes 4 items (eg, “I would recommend it to a friend”), while the ease-of-use scale consists of 9 items. Originally, the ease-of-use subscale contained 11 items (eg, “Using it is effortless”); however, items 7 and 8 were excluded due to low factor loadings (≤0.20) [45]. Gao et al [46] reported that the satisfaction and ease-of-use subscales had high internal consistency (αs from 0.88 to 0.95). Participants are also asked one open-ended question, inviting them to share any additional thoughts or experiences with the Time2Feel app.

#### Subjective Emotion Experiences

During the 7 days of the EMA and ambulatory monitoring period, families report on their emotion experiences using the Time2Feel app. Resulting variables include the specific emotion experience, the intensity with which the emotion is experienced, the activity the family member is engaged in at the time, and any individuals the family member was with at the time of the survey. Over the 7-day period, each family member’s reported emotions are categorized as positive or negative, and their intensity ratings are used to calculate overall positive and negative affect scores. These scores are used to estimate family-wide emotion dynamics.

#### Physiological Arousal

HR and EDA are continuously recorded during study hours throughout the 7-day EMA period using the Empatica E4 wristband. Changes in blood volume pulse are used to estimate HR, while skin conductance levels are derived from EDA. Data are imported into AcqKnowledge version 5.0, and guidelines for quantifying HR and EDA are followed [36,47]. Physiological data are synchronized with EMA survey responses to examine physiological states during self-reported emotional experiences. For both HR and EDA, time-series data are segmented into 10-second increments using AcqKnowledge 5.0. Intervals within ±5 minutes of survey responses are averaged. This procedure results in 28-56 HR and EDA data points per family member, which are used to estimate family-wide emotion dynamics. These physiological indicators contribute to our understanding of how emotional states are experienced and coregulated across family members in daily life, addressing the study’s primary aim of capturing the emotional processes that influence children’s psychological functioning.

#### Family-Wide Emotion Dynamics

Family-wide emotion dynamics are indexed through two primary variables derived from the family emotion networks (see analytic plan): density and centrality [31].

Density is a measure of the overall connectivity of the network, reflecting how closely family members are linked through emotional and physiological states. It is calculated by averaging the strengths of all connections between family members. Each connection, or “edge,” represents the strength of the shared emotional and physiological state between two members. Each family will receive a positive and negative emotion density score, representing the intensity of these emotions and their physiological states. Research highlights the significance of density, showing that denser networks are more ingrained and may therefore be more resistant to change [48]. Higher density scores indicate stronger and more consistent connections between family members’ emotions.

Centrality is a measure of how central, or important, each family member is within the broader family network. It is calculated by summing the in-strength (ie, the influence from other family members toward the individual) and the out-strength edges (ie, the influence exerted by the individual toward other family members). Each family member will have a centrality score, which will indicate how impactful this family member is within the whole family. A higher centrality score suggests greater importance.

### Secondary Outcomes: Family, Parent, and Child Variables

#### Family Functioning

Parents’ beliefs about their families’ strengths, abilities, and competencies in managing challenges and maintaining healthy functioning are assessed using the Family Functioning Styles Scale (FFSS) as part of the pre-EMA questionnaires [49]. The FFSS asks parents to indicate to what extent their family is characterized by 26 different qualities on a 5-point scale ranging from not at all like my family to almost always like my family (eg, “we generally agree about how family members are expected to behave”). The FFSS has been reported to have high internal consistency [49]. This measure is included to assess patterns of family functioning that may shape family-emotion networks, directly supporting our secondary objective of identifying family-level characteristics that influence the emotional dynamics observed during the EMA period.

#### Parent Emotion Socialization

Parents complete the Coping with Children’s Negative Emotions Scale (CCNES) during the pre-EMA questionnaires [50]. This is a self-report measure that evaluates how parents respond to their children’s negative emotions. The CCNES presents 12 vignettes describing hypothetical situations in which their child expresses negative emotions (eg, “if my child falls off his/her bike and breaks it, and then gets upset and cries, I would...”). Parents rate the likelihood of each response on a 7-point scale (1=very unlikely to 7=very likely) across six response types. These responses are categorized into six subscales: Minimizing Reactions, Punitive Reactions, Distress Reactions, Expressive Encouragement, Problem-Focused Reactions, and Emotion-Focused Reactions. Consistent with previous research, we use a version with the six response types combined into two overall scores: Supportive practices (Expressive Encouragement, Problem-Focused, and Emotion-Focused reactions) and Unsupportive practices (Punitive, Minimizing, and Distress reactions) [51]. This measure demonstrates good internal consistency and test-retest reliability, along with sufficient construct validity [50]. The CCNES is used to assess whether parent responses to children’s negative emotions contribute to family emotion dynamics and influence child outcomes.

#### Parent Gendered Emotion Beliefs

Parents complete the Parents’ Gendered Emotion Beliefs Scale (PGEB) during the pre-EMA questionnaires [52]. The PGEB is a 15-item questionnaire that assesses parents’ gendered beliefs about emotion expression, gender-neutral beliefs, and gendered emotion socialization (eg, “I don’t think boys should cry because it shows weakness”). Responses are rated on a 4-point scale, from strongly disagree to strongly agree. The PGEB has shown good internal consistency for both the overall scale and each of the three subscales [52]. The PGEB is used to examine how parents’ gendered emotion beliefs may shape family emotion patterns.

#### Parent Emotion Regulation

Parents self-report on how often they engage in six emotion regulation strategies to manage negative emotions on the Regulation of Emotion Systems Survey (RESS) during the pre-EMA questionnaire [53]. This measure consists of 48 items using a 5-point scale ranging from never to always. Each of the six strategies—distraction, rumination, cognitive reappraisal, suppression, engagement, and relaxation—is assessed by 8 items each. Each subscale demonstrates high internal reliability [53]. Understanding parents’ regulatory practices supports the objective of identifying individual and family-level factors that contribute to family emotion dynamics and child psychological outcomes.

#### Parent Negative Emotionality

In the pre-EMA questionnaire, parents report on their tendency to react frequently and intensely to negative emotions, adapted from the 7-item Child and Adolescent Disposition Scale (CADS) [54]. The measure is altered to have parents reflect on their own emotional behavior over the past 12 months and indicate how often negative emotionality occurred (eg, “moods change unpredictably”), using a scale from 4-point scale (not at all to very much/very often). The CADS questionnaire shows high internal consistency [54]. This measure is used to identify how parent emotionality influences child outcomes (eg, child emotionality and psychopathology).

#### Parent Psychopathology

In the demographics section of the pre-EMA questionnaire, parents are asked if they currently have a diagnosis for a psychological condition, and if so, what they have been diagnosed with, and at what age they were diagnosed. They are then asked to rate the severity of their condition from not severe—I rarely or do not experience any symptoms to extremely severe—I always experience symptoms*.* Additionally, parents fill out the Kessler 10 (K-10) during the pre-EMA questionnaire, which assesses 10 symptoms of anxiety and depression (eg, “how often did you feel tired out for no good reason?”) on a 5-point Likert scale (none of the time to all of the time) [55]. The K-10 had been found to be a reliable measure in assessing symptomology in adults [56]. Parent psychopathology is assessed to support the study’s goal of identifying parent-level factors that shape family emotion networks and child outcomes.

#### Perceived Parental Stress

Parents respond to the 17-item Parental Stress Scale (PSS) during the pre-EMA questionnaires [57]. The PSS measures perceived stress related to parenting (eg, “In the last month, how often have you been upset because of something that happened unexpectedly?”) on a 5-point scale (strongly disagree to strongly agree). Adequate internal consistency of the PSS was found across a range of samples [57]. Assessing parental stress supports the study’s aim of identifying parent-level factors that may shape family-emotion networks and influence children’s emotional functioning.

#### Child Emotion Regulation

Child emotion regulation is measured through parent-report, child self-report, and a computerized CRT. These measures are described below.

Parents complete the RESS questionnaire for the focus child during the pre-EMA questionnaire [53]. Focus children also self-report their own strategy use during the pre- and post-EMA questionnaires.

Parents report on the focus child’s self-regulation and ability to manage their emotions using the Emotion Regulation Checklist (ERC) at pre- and post-EMA. The ERC is a 24-item parent-report measure consisting of two subscales: emotion regulation and emotional lability and negativity [58]. Items are rated on a 4-point scale (rarely or never to almost always). The ERC has been validated for children aged 6 to 12 years and demonstrates acceptable internal consistency for both subscales [58].

The focus children’s effectiveness in using cognitive reappraisal, an emotion regulation strategy known to reduce negative affect, is measured by their scores on the CRT [38]. This task is completed on Inquisit Web (Millisecond Software), a web-based platform, during the training session. In this task, children are trained to look and think about negative and neutral images of people and objects in different ways. When shown the “look” cue, children are instructed to observe the images naturally, without attempting to reappraise their emotions. When shown the “far” cue, children are trained to reappraise their emotions by either imagining the image as being far away from them or by imagining the image as being unreal. Following the cue and the image, children rate their negative emotion intensity on a feelings thermometer ranging from 0=felt no negative emotion to 3=felt a big amount of negative emotion. This task is comprised of a practice phase followed by a test phase consisting of 45 negative or neutral images, divided into three blocks with breaks in between. Image and cue types are counterbalanced and randomized across trials, resulting in three trial conditions: far negative (reappraisal), look negative (nonregulation), and look neutral (nonemotional). Cognitive reappraisal effectiveness is calculated as the percentage of negative affect reduced by reappraisal on far-negative trials compared to look-negative trials ([Look Negative Rating–Far Negative Rating]/Look Negative Rating)×100).

Child emotion regulation measures, including the RESS, ERC, and CRT, are used to examine how aspects of family functioning and parent characteristics (eg, psychopathology, emotionality, and perceived stress) relate to children’s emotion regulation, a key component of psychological functioning and well-being.

#### Child Negative Emotionality

During the pre- and post-EMA questionnaires, both parents and the focus child report on the focus child’s propensity to react frequently and intensely to negative emotions on the 7-item CADS [54]. They are asked to reflect on the child’s emotional behavior and indicate how often these behaviors occurred in the past 12 months (eg, “moods change unpredictably”) using a scale from 1=not at all to 4=very much/very often. The CADS presents acceptable internal consistency for both parent-report and child self-report [54]. Additionally, the CADS is used as an outcome measure to examine whether family emotion dynamics are associated with changes in children’s emotion reactivity over time, supporting the study’s aim of understanding how family-level processes relate to children’s emotional functioning.

#### Child Psychopathology

In the demographic section of the pre-EMA questionnaire, parents are asked if either of their children that are participating in the study currently have a diagnosis for a psychological condition and if so, what have they been diagnosed with, at what age were they diagnosed and then asked to rate the severity of their condition from 0=not severe—he/she rarely or does not experience any symptoms to 3=extremely severe—he/she always experiences symptoms*.*

Parents report on the focus child’s behavior and the focus child self-reports on their own behavior using the Brief Problems Monitor (BPM) as part of the pre- and post-EMA questionnaires [58]. The BPM includes 19 items rated on a scale from 0=not true to 3=very true, which measures internalizing problems (eg, “feels worthless or inferior”), attention problems (eg, “acts too young for his/her age”), and externalizing problems (eg, “argues a lot”). The internalizing and attention subscales each consist of six items, while the externalizing subscale contains seven items. Subscale scores are calculated by summing item ratings within each domain. A total problems score is computed by summing all 19 items. Both subscale and total scores are converted to standard T scores based on age- and gender-specific norms. T scores below 65 are considered within the normal range, while scores above 65 indicate clinically elevated concerns. Each subscale has been shown to display acceptable test-retest reliability correlations and internal consistencies [59]. Measures of psychopathology are included as outcome variables to examine how family emotion dynamics relate to children’s emotional functioning, directly addressing the study’s primary objective.

### Statistical Methods

#### Sample Size

Simulations in triadic family network models with as many as 12 nodes demonstrate reliable network estimation with at least 59 families and as few as 56 time points [33]. Nodes are observed variables in the network, such as mother positive emotion, father positive emotion, child positive emotion, sibling positive emotion, mother HR, and father HR. Fewer nodes would require fewer families and timepoints. Published benchmarks indicate that temporal networks require more than 75 time points to achieve reliable precision and sensitivity and can accommodate up to 25% missing data [60]. The FACES study design provides between 28 and 56 time points per primary outcome for each family member with an estimated sample size of 70 families, or 280 individual family members. This design considers both data requirements and the need to minimize participant burden for children as young as 7 years [60].

#### Analytic Plan for Primary and Secondary Outcomes

To address study aims, the analytic plan involves a network analytic approach, specifically multilevel vector autoregressive modeling using the mlVAR package in R. The analytic plan includes three stages: (1) preparing the data, (2) estimating networks and calculating the primary outcomes (ie, network density and centrality outcomes), and (3) examining their relation with secondary outcomes (eg, individual emotion-regulation outcomes). This plan aligns with analytical approaches recommended by the developers of these methods and prior applications of network analysis to dyads and triads [33,61-63].

#### Data Preparation and Assumptions Testing

Descriptive statistics for all variables, compliance with EMA surveys and ambulatory physiology wear times, and mean satisfaction with the Time2Feel app (assessed via the Usefulness, Satisfaction, and Ease of Use questionnaire) are to be reported.

In terms of family emotion networks, mlVAR assumptions include equal time spans between timepoints (eg, EMA surveys), multivariate normality, and stationarity. To accommodate our pediatric sample, the FACES protocol features equal spacing within designated periods (eg, hourly from 4 PM to 8 PM on weekdays and 10 AM to 6 PM on weekends) but a longer overnight interval between the last survey of one day (eg, 8 PM) and the first survey of the next (eg, 4 PM). To address this, the network model excludes estimates of relationships between evening and morning surveys. Normality is assessed using the Kolmogorov-Smirnov test. Data are detrended to ensure stable means, variances, and autocorrelations over time. Then the Kwiatkowski-Phillips-Schmidt-Shin unit root test is used to examine stationarity [31]. In instances that stationarity assumptions are not met, a semiparametric time-varying vector-autoregressive model is used instead [64].

Secondary outcomes (ie, RESS and CADS) are analyzed using multiple regression. Their distribution and normality are assessed by examining skewness and kurtosis, and outliers are identified as data points that deviate more than 3 SDs from the sample mean. Identified outliers are winsorized [65].

#### Network Estimation and Family-Wide Emotion Dynamics

**Figure 2:**
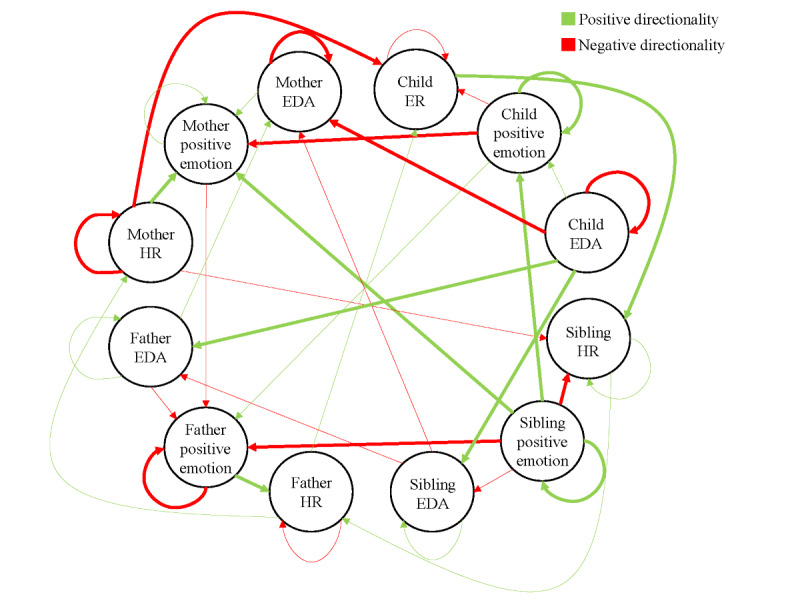
Family-wide positive emotion network. This network represents positive emotion-physiology dynamics within families. Each node reflects a family member’s positive emotion, HR, or EDA, resulting in 12 nodes per network. Edges show the effect of one node on another, controlling for all other nodes. Thicker edges indicate stronger effects. Green edges represent excitatory (positive) effects and red edges represent inhibitory (negative) effects. EDA: electrodermal activity; ER: emotion regulation; HR: heart rate.

**Figure 3:**
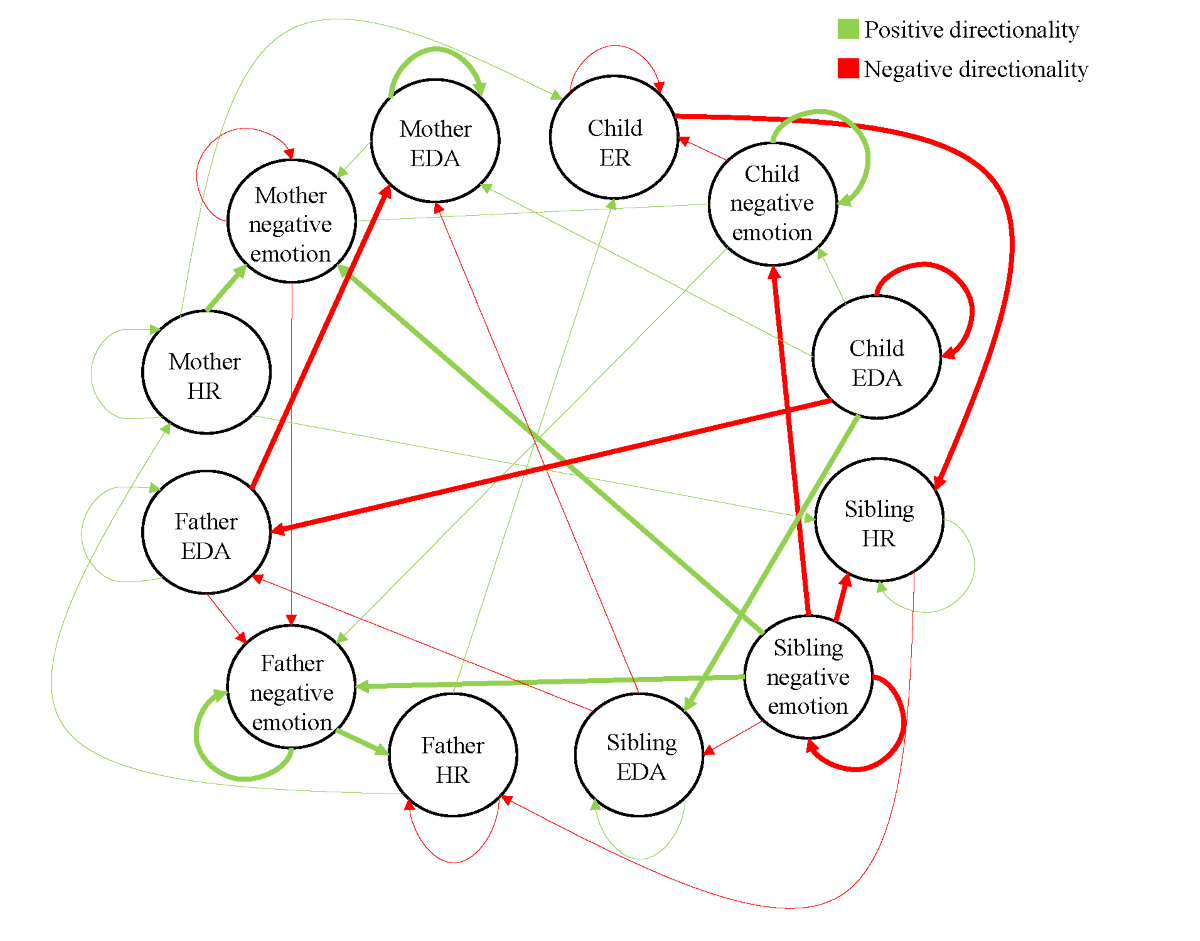
Family-wide negative emotion network. This network represents negative emotion-physiology dynamics within families. Each node reflects a family member’s negative emotion, HR, or EDA, resulting in 12 nodes per network. Edges show the effect of one node on another, controlling for all other nodes. Thicker edges indicate stronger effects. Green edges represent excitatory (positive) effects and red edges represent inhibitory (negative) effects. EDA: electrodermal activity; ER: emotion regulation; HR: heart rate.

Temporal nomothetic (group-level) and idiographic (family-specific) networks are estimated using the mlVAR package and visualized with the graphicalVAR package in R [66,67]. These analyses produce Family-Wide Emotion Networks as shown in Figures 2 and 3. Figure 2 represents positive emotion-physiology dynamics within families, and Figure 3 represents negative emotion-physiology dynamics. Each node (circle) represents one of three indicators—emotion experience (positive or negative), HR, or EDA—for each of the four family members, resulting in 12 nodes per family (3 measures×4 individuals). The edges, or lines connecting the nodes, represent the effect of one family member’s state on another while accounting for the influence of other family members in the network [31]. Thicker edges indicate stronger effects, which can be excitatory (green) or inhibitory (red). Nomothetic networks show average temporal associations across all families and are used to describe group-level patterns [35]. Idiographic networks, which represent how emotions and physiological states interact within individual families over time, are used to calculate metrics of density and centrality.

Two family emotion density scores are calculated: one for positive emotion and one for negative emotion. The model also calculates in-strength and out-strength edges for each node. In-strength shows how much a node is influenced by others, while out-strength indicates how much it influences other nodes. Using these measures, eight centrality scores are calculated—one for each family member in both the positive and negative emotion networks. The reliability and significance of edges and centrality measures are assessed using CIs and bootstrap methods [66].

#### Family-Wide Emotion Dynamics and Secondary Outcomes

To test the primary objective of this protocol—how family emotion networks contribute to children’s functioning—multiple regression analyses are conducted in which child emotion regulation (ie, RESS, ERC, and CRT) and child psychopathology (ie, BPM) are regressed on family-wide density scores. To examine the secondary aim—identifying which family, parent, and child factors may influence family emotion networks—additional regression analyses are conducted in which family-wide density scores are regressed on family (ie, FFSS), parent (ie, CCNES, PGEB, RESS, CADS, K-10, and PSS), and child (eg, CADS) factors. To disentangle the contributions of specific family members to child outcomes, regression analyses are conducted in which child emotion regulation (ie, RESS, ERC, and CRT) and child psychopathology (ie, BPM) are regressed on each family member’s centrality score. This approach will clarify the specific contributions of individual family members to these child outcomes, accounting for their relative prominence and connectivity within the family emotion network.

### Methods for Additional Analyses

Based on prior research demonstrating that real-time self-reporting of emotional experiences may support improvements in children’s functioning, additional analyses include applying repeated-measures ANOVAs to test for changes in child emotion regulation (ie, RESS and ERC), child emotion reactivity (ie, CADS), and child psychopathology symptoms (ie, BPM) from pre- to post-EMA. If changes are detected, mediation analyses are then applied to test whether family-wide emotion dynamics variables account for the observed differences.

### Missing Data Analyses

Based on prior research using EMA designs, an average of 25% missing data is expected [39]. Patterns and reasons for missingness will be examined using descriptive statistics and Little’s Missing Completely at Random test. If the missing-at-random assumption is met and the amount of missing data is within the expected range, appropriate imputation methods are applied. For missing primary outcomes, including EMA survey responses and ambulatory physiology data, the Kalman filter is applied, as it accounts for the nested and time-series nature of the data [68,69]. For missing secondary outcomes, such as parent and child questionnaire data, multiple imputation is used to prevent biased parameter estimates associated with pairwise or listwise deletion [68,69]. Rates of missing data are to be reported.

### Interim Analysis

There are no plans for interim data analysis.

### Data Monitoring, Management, and Dissemination

Monitoring of data is completed by the research team on an ongoing basis.

#### Protocol Amendments

All protocol amendments are submitted to the University’s Research Ethics Board for review and approval prior to implementation.

#### Harms

Any adverse events are to be reported to the University’s Research Ethics Board as per ethical guidelines.

#### Auditing

Audits are conducted as required by the sponsoring institution’s Research Ethics Board.

#### Authorship Eligibility Guidelines

Authorship eligibility will be determined based on substantial contributions to study conceptualization, design, analysis, and writing, based on the International Committee of Medical Journal Editors. Contributors who do not meet authorship criteria will be acknowledged. No professional medical writers will be used in the preparation of study-related publications.

#### Dissemination Policy

The findings from this study will be disseminated through multiple channels to maximize visibility and impact within the scientific and broader public communities. Key dissemination methods will be through peer-reviewed publications, presentations are national and international conferences, and media outlets (eg, the lab’s official website, news interviews, and magazines).

## Results

The FACES study was funded by the Social Sciences and Humanities Research Council of Canada via an Insight Grant (SSHRC #435-2020-0908) and the Natural Sciences and Engineering Council of Canada via a Discovery Grant (NSERC # RGPIN-2020-05855) as of April 2020. Data collection began in September 2022. As of May 2025, the FACES study has recruited and collected data from 48 eligible families. Data collection will continue until March 2026. Data analysis will begin after March 2026, with results expected to be published in Fall 2027.

## Discussion

### Overview

FACES presents an innovative protocol for examining day-to-day emotion dynamics within entire family systems. By combining questionnaires, real-time emotion tracking, ambulatory physiology monitoring, and advanced analytical methods, this approach captures the interconnected nature of family-wide emotional processes. Exploring emotion dynamics across the entire family unit addresses a major gap in the emotion and family literature, which up until now has heavily focused on individual experiences of emotion or emotion interactions between dyads (primarily mother-child relationships) [8,70]. Considering the whole-family system is essential for understanding children’s emotional development and how emotions are influenced by the broader family context, this protocol was developed with two key objectives in mind: (1) to explore how family emotion networks influence children’s emotional functioning and development, and (2) to identify family, parent, and child factors (eg, parental psychology and child emotion regulation abilities) that influence these emotion networks. This protocol serves as a valuable framework for future researchers exploring family-wide emotion dynamics.

### Methodological Innovations

To our knowledge, combining EMA and ambulatory physiological monitoring across entire family units has never been explored, likely due to past limitations in technology and analytical methods. Recent advancements in technology and analytic approaches have made such research feasible. This protocol not only balances ecological validity by capturing emotional experiences in individuals’ everyday contexts but also allows for a comprehensive assessment of emotion across experiential and physiological levels, within whole-family units. By pairing EMA emotion reports and ambulatory physiological monitoring, this protocol allows for a better understanding of how family members can influence each other’s emotional states across multiple levels of measurement. This approach enables researchers to assess emotion synchrony, spill-over, and contagion across the whole family unit, recognizing that interactions extend beyond dyadic relationships to encompass more complex family dynamics. Furthermore, the short-term longitudinal design allows for data collection across diverse contexts while minimizing participant burden. Conducting the study in participants’ natural environments, outside of regular business hours, and over the course of a week enables repeated and nested data collection without being too overwhelming for parents and children. Following recommendations from Sosa-Hernandez et al [30], this protocol implements participant training on study equipment, regular check-ins, and bonus remuneration to enhance compliance and participant satisfaction. Collectively, these features ensure a robust, ecologically valid assessment of emotional dynamics within family systems.

### Analytical Contributions

Datasets generated from this protocol involve numerous data points collected at multiple levels of measurement across several family members. This structure results in nested data, where measurements are interdependent due to shared family contexts and repeated measurements. Our study advances the literature by looking at critical relationships within the family (influence of parents and siblings), as well as through multimethods (eg, subjective emotion reports and corresponding physiology). To appropriately model these complex emotion exchanges, we propose a network analytic approach, specifically multilevel vector autoregressive modeling [33]. The mlVAR approach accounts for the temporal and nested nature of family emotion data by capturing how emotions expressed by one family member influence and are influenced by others over time. This method provides key metrics for understanding family-emotion dynamics, such as density and centrality. Overall, these advanced statistical methods allow researchers to explore complex family-emotion dynamics, capturing how emotions change and stabilize within the family unit.

### Limitations and Future Directions

Though this protocol serves as a guiding framework for researchers conducting family-wide emotion research, certain limitations must be acknowledged. The FACES protocol is the first of its kind to study family dynamics through a multilevel approach that examines both experiential and physiological aspects of emotion across family units of four. However, this protocol currently focuses solely on typical family structures consisting of two parents and two children and does not currently account for more complex family systems (eg, families with additional children or extended family members). Future research could be expanded to include these more complex and interdependent family configurations to better reflect the diversity and interconnected nature of families beyond the immediate household.

Although this protocol goes beyond subjective reports by incorporating physiological data, it does not account for behavioral or vocal indicators of emotion experiences**.** Expanding the protocol to include behavioral measures would enrich the understanding of emotional exchanges. Additionally, the Time2Feel app used in this study captures only singular emotions, limiting the ability to reflect complex emotional experiences that involve co-occurring emotions. Future adaptations could include options for reporting multiple simultaneous emotions to provide a more realistic depiction of emotional experiences.

This protocol includes four data collection points per day and restricts measurements to specific hours of the day (eg, after school or work hours). This limited-time sampling may not fully capture the breadth of daily emotional dynamics and could omit key moments of emotional variability. However, additional sampling must be balanced with participant compliance, especially when involving children, as research suggests they generally have lower compliance rates than adults [30]. Additionally, many children are not allowed to have phones during school, which is a limiting factor. Future protocols could consider increasing the number of daily measurement points and diversifying sampling times to provide a more comprehensive assessment of emotion throughout the day while also implementing strategies to improve compliance.

Compared to laboratory studies, managing technology in a home-based setting can be particularly challenging, as researchers cannot be physically present to troubleshoot issues. To address this, researchers are encouraged to provide adequate training on equipment and remain available to connect with participants digitally to troubleshoot. [30]. One significant challenge with ambulatory physiological monitoring is the increased risk of data loss due to motion artifacts [71]. Unlike studies measuring stationary physiology, ambulatory measurements of HR and EDA are susceptible to motion artifacts, which can render the data unusable or require extensive cleaning. To address this challenge, several studies provide guidelines for managing and cleaning datasets affected by motion artifacts [30,71]. Current wearable devices for ambulatory monitoring remain limited in their ability to manage motion artifacts. While the Empatica E4 wristband performs comparably to the “gold-standard” ECG when measurements are taken in a stationary setting, its data quality declines during ambulatory monitoring due to increased susceptibility to motion-related interference [40]. As wearable technology continues to evolve, future research protocols should prioritize devices better equipped to handle ambulatory assessment, ensuring more accurate and reliable physiological data collection in real-world settings.

Despite these limitations, the FACES protocol has many strengths and offers a unique and comprehensive method for evaluating family-wide emotion dynamics. This protocol holds promise for enhancing family emotion science.

## Appendix

Multimedia Appendix 1 Completed SPIRIT (Standard Protocol Items: Recommendations for Interventional Trials) checklist for the FACES study protocol.

## Abbreviations

BPM: Brief Problems Monitor
CADS: Child and Adolescent Disposition Scale
CCNES: Coping with Children’s Negative Emotions Scale
CRT: cognitive reappraisal task
ECG: electrocardiogram
EDA: electrodermal activity
EMA: ecological momentary assessment
ERC: emotion regulation checklist
FACES: Family and Child Emotion Study
FFSS: Family Functioning Styles Scale
HR: heart rate
K-10: Kessler 10
PGEB: Parents’ Gendered Emotion Beliefs Scale
PPG: photoplethysmography
PSS: Parental Stress Scale
RESS: Regulation of Emotion Systems Survey
SPIRIT: Standard Protocol Items: Recommendations for Interventional Trials

## Data Availability

Data sharing is not applicable to this article as no data sets were generated or analyzed during this study.
